# Roles of db-cAMP, IBMX and RA in Aspects of Neural Differentiation of Cord Blood Derived Mesenchymal-Like Stem Cells

**DOI:** 10.1371/journal.pone.0009398

**Published:** 2010-02-24

**Authors:** Murni Tio, Kian Hwa Tan, Wendy Lee, Ting Ting Wang, Gerald Udolph

**Affiliations:** Biomedical Science Institutes, Institute of Medical Biology, Singapore, Singapore; Institut de la Vision, France

## Abstract

Mesenchymal stem cells (MSCs) have multilineage differentiation potential which includes cell lineages of the central nervous system; hence MSCs might be useful in the treatment of neurodegenerative diseases such as Parkinson's disease. Although mesenchymal stem cells have been shown to differentiate into the neural lineage, there is still little knowledge about the underlying mechanisms of differentiation particularly towards specialized neurons such as dopaminergic neurons. Here, we show that MSCs derived from human umbilical cord blood (MSC^hUCBs^) are capable of expressing *tyrosine hydroxylase* (*TH*) and *Nurr1*, markers typically associated with DA neurons. We also found differential phosphorylation of TH isoforms indicating the presence of post-translational mechanisms possibly activating and modifying TH in MSC^hUCB^. Furthermore, functional dissection of components in the differentiation medium revealed that dibutyryl-cAMP (db-cAMP), 3-isobutyl-1-methylxanthine (IBMX) and retinoic acid (RA) are involved in the regulation of *Nurr1* and *Neurofilament-L* expression as well as in the differential phosphorylation of TH. We also demonstrate a possible inhibitory role of the protein kinase A signaling pathway in the phosphorylation of specific TH isoforms.

## Introduction

Stem cells with their intrinsic capacity to self-renew and differentiate have sparked great interest as potential tools in cellular replacement therapies in neurodegenerative diseases. Although most clinical applications of stem cells will likely have to rely on *in vitro* expansion and differentiation strategies prior to cell transplantation, the knowledge and methods of differentiating stem cells into specialized cell types is still a major limitation.

In Parkinson's disease (PD), dopaminergic (DA) neurons are progressively lost, and this loss is causative for the severe clinical symptoms of PD. DA neurons are characterized by the expression of tyrosine hydroxylase (TH), the rate-limiting enzyme in the biosynthesis of dopamine. A conceivable treatment option for PD could be to utilize stem cells or their derivatives to attempt functional replacement of lost DA neurons. Many potential sources of stem cells have been described including MSCs from the peripheral blood, bone marrow, adipose tissue and umbilical cord blood. It has been reported that multipotent MSCs are capable of differentiating into a variety of cell types from different germ layers [1], [2], [3], [4], [5], [6], [7]. The major advantage of umbilical cord blood derived cells is their relative abundance as they can be obtained easily and non-invasively post delivery of newborns. Moreover, these cells can be cryo-preserved hence they are available for autologous transplantation even years after harvesting.

Mesenchymal-like stem cells derived from the human umbilical cord blood (MSC^hUCBs^) were demonstrated to express neural markers following exposure to induction media which included RA, IBMX and db-cAMP [4], [5], [6], [7]. IBMX and db-cAMP elevate intracellular cAMP levels thereby possibly activating protein kinase A (PKA) and it was demonstrated recently that the PKA pathway is a crucial mediator of neural differentiation of MSC^hUCB^ [6].

MSCs from the bone marrow and cord blood are able to upregulate genes associated with DA neurons such as the orphan nuclear receptor Nurr1 and TH [5], [8] Recently, it was shown that cord blood derived MSCs could differentiate into neurons with functional properties such as the ability to synthesize and release dopamine into the culture supernatant upon stimulation [7]. Nurr1 is required for the generation of midbrain dopaminergic neurons [9], possibly through the regulation of TH expression [10]. Nurr1 heterodimerizes with retinoid X receptors (RXR) and such dimers can act as transcriptional modulators by binding to retinoic acid-responsive elements in target gene promoters [11]. Together, these suggest a possible involvement of RA in the regulation of TH expression.

Here, we report that MSC^hUCB^ can be induced to adopt neural-like features. They upregulate general neural markers and TH, the gold standard marker for DA neurons as well as its various phosphorylated isoforms. We also found that the expression of CD133 [12], a marker typically associated with stem cells was abolished following differentiation. To study the roles of individual components in the complete differentiation medium we carried out dissection analysis of the medium. We found that IBMX and db-cAMP induced neural-like morphology as well as *Nurr1* expression. IBMX and RA acted synergistically to upregulate *NF-L* expression. RA and db-cAMP increased levels of distinct phosphorylated TH (P-TH) isoforms. Furthermore, blocking the PKA signaling pathway resulted in predominant phosphorylation of at least one TH isoform. Our results indicate that phosphorylation of TH isoforms is differentially regulated, presumably via different signaling cascades involving RA and cAMP. We also propose an inhibitory role of PKA in TH phosphorylation.

## Materials and Methods

### Isolation of MSC^hUCBs^

Cord blood samples were received from the Singapore Cord Blood Bank. Isolation of MSC^hUCBs^ was done as described elsewhere [4] and the MSC populations used in this study were derived from three individual cord blood donors. Briefly, the mononuclear cell fraction was separated by Ficoll (Biochrom) gradient followed by lysis of RBCs with ammonium chloride. The cells were then seeded in MyeloCult medium (Stem Cell Technologies) supplemented with 1×10^−7^ M dexamethasone (Sigma). Adherent cells were trypsinized (0.125% Trypsin-EDTA) and grown for further studies.

### Cell Propagation and Neural Differentiation

MSC^hUCBs^ were grown in growth medium consisting of Dulbecco's Minimum Essential Medium (DMEM, Sigma) with low glucose (1,000 mg/L) and supplemented with 20% Fetal Calf Serum (FCS, Hyclone) and 100 U/ml penicillin/streptomycine (DL20). Incubation was done in a humidified incubator equilibrated with 5% CO2 (37°C). For induction experiments, growth medium was replaced by cytokine induction medium (CIM) consisting of DMEM with low glucose and supplemented with 5% FCS and antibiotics (DL5), 0.5 mM 3-isobutyl-1-methylxanthine (IBMX; Sigma), 10^−5^ M retinoic acid (RA; Sigma), 1 mM dibutyryl-cAMP (db-cAMP; Sigma), 50 ng/ml nerve growth factor-β (NGF-β; Sigma) and 20 ng/ml basic fibroblast growth factor (bFGF; Pepro-Tech) or as otherwise indicated [4], [7]. For media dissection experiments, the same concentrations of components were used. Change of media was done every three days. For quantification of cells with neurite extension, 8 to 15 random frames were selected for cell counting. For consistency of results, cell culture and induction experiments were generally carried out in triplicates. To ensure that the observed results were not obtained from just one particular cord blood unit, a total of three cord blood units were investigated. For all MSC populations, the experiments were carried out using cells from passages 5–8.

### Western Blotting and Immunocytochemistry

Protein extraction was carried out using Reagent 3 from ReadyPrep sequential extraction kit (BioRad). After trypsinization, cells were gently washed twice with PBS and re-suspended in Reagent 3. They were then exposed to three rounds of freeze (liquid nitrogen) - thawing (37°C water bath) procedures, followed by centrifugation to separate cell debris from the protein extracts. Protein concentrations were read using the standard Bio-Rad Essay. 50 µg of total cell extracts were fractionated by SDS-PAGE and transferred onto nitrocellulose membranes (Hybond-C, Pharmacia). Transfer was done at 4°C for four hours at 100 V. The membranes were then blocked with 5% non-fat milk powder, rolled overnight at 4°C, incubated with primary and secondary antibodies at room temperature for two hours each, followed by six washes with PBT (PBS +0.05% Tween-20) after each antibody incubation. Signals were detected using Super-Signal West Pico Chemiluminescent substrate (Pierce).

For immunocytochemistry, cells were grown in DL20 on cover slips in the 24-well plates until 80% confluency, then exposed to neural induction cocktails/components (in DL5) for 3 days and fixed with 4% paraformaldehyde. For the control, DL20 was changed to DL5 (without any induction components). The fixed cells were washed with PBT, blocked with 3% BSA followed by antibody incubation and analysis using confocal microscope. Topro-3 was used to label DNA.

For Western Blotting and Immunocytochemistry, the following primary antibodies were used: mouse α-Tau (Chemicon, 1∶500), mouse α-Neuronal Specific Enolase (NSE, Chemicon, 1∶1,000), rabbit α-Glial-Fibrillary-Acidic-Protein (GFAP, Chemicon, 1∶500), rabbit α-Tyrosine Hydroxylase (Sigma, 1∶1,000), rabbit α-Phospho-Tyrosine Hydroxylase (Cell Signaling, 1∶2,000), mouse α-Phospho-p44/42 MAPK (Thr 202/Tyr 204) (E10) (Cell Signaling, 1∶2,000), mouse α-β-actin (Sigma, 1∶1,000), mouse α-Synaptophysin (Sigma, 1∶100). Secondary antibodies used were Cy3- and FITC- conjugated goat anti-rabbit and goat anti-mouse antibodies. For the staining controls, the cells were incubated with just the secondary antibodies. For inhibition experiment, H89 (LC Laboratory) was used at 20 µM. Cells were grown in DL20 until 80% confluency, then exposed to CIM plus H89 (in DL5) for 1 day before the proteins were extracted.

### FACS Analysis

Cells were washed with PBS, harvested with trypsin treatment and resuspended in 10% FBS in PBS. Harvested cells were incubated with PE conjugated anti-human CD133 (Miltenyi Biotech, 1∶20) or isotype control for 15 min on ice, washed, resuspended in 10% FBS in PBS and analyzed using FACS Calibur (BD). Cells were deemed positive for CD133 when the average geometric mean fluorescence intensity (GMFI) from 3 individual experiments was significantly (p<0.05) increased as compared to that of the respective isotype controls.

### RT-PCR

Cells were washed with PBS, harvested with trypsin treatment and pelleted. Total mRNAs were extracted using either the TRIzol kit (Invitrogen) or the RNeasy Mini Kit (Qiagen) according to the manufacturers' instructions. The concentrations of the extracted RNAs were determined by measuring the absorbances at 260 nm (A260) using a spectrophotometer. Only RNA samples of high quality which had the A260/A280 ratios of between 1.9 and 2.1 were used. 2 µg of total RNA was used in each reverse transcription reactions in a total of 20 µl reaction volume. The first strand cDNA was synthesized by incubation in MulV RT Enzyme (New England Biolabs) at 37°C for 1 hour, followed by heat inactivation at 90°C for 5 minutes. An equivalent of 0.1 µg of template was used in each subsequent PCR reactions. For the negative controls, no templates was used for each primer sets. β-actin was used as the loading control. Primers used were: **NSE** (F-CCTTGAGCAGCAGACAGTTGC, R-TTGGAGCTGGTGAAGGAAGCC), **NF-H** (F-GAGAAGAAGGAACCTGCTGTCG, R-CTTCTCTGGAGGCTTGCTGTCT), **NF-M** (F-TGGGAAATGGCTCGTCATTT, R-CTTCATGGAAACGGCCAATT), **NF-L** (F-TCCTACTACACCAGCCATGT, R-TCCCCAGCACCTTCAACTTT), **GFAP** (F-GGAGGGGACGCTGGTAGAGAT, R-TGCAGGAATATGAGCCAGTGTC), **Nurr-1** (F-CACTACGGCGTGCGCACC, R-GCTAATCGAAGGACAAACAG), **TH** (F-TCATCACCTGGTCACCAAGTT, -GGTCGCCGTGCCTGTACTR), **DβH** (F-GTGACCAGAAGGGGCAGATCC, R-GGCCGGCTTCCTCTGGGTAGT) and **β-actin** (F-TCTACAATGAGCTGCGTGTG, R-CAACTAAGTCATAGTCCGCC). For all experiments, 35 cycles of PCR were carried out. Unless otherwise stated, experiments were generally conducted in triplicates. All RNA extractions for RT-PCR experiments were carried out at day 1 post-induction. The DNA bands were quantified using the Image J program and all the experimental results were normalized towards β-actin.

## Results

### Neural Differentiation of MSC^hUCB^

**Figure 1:**
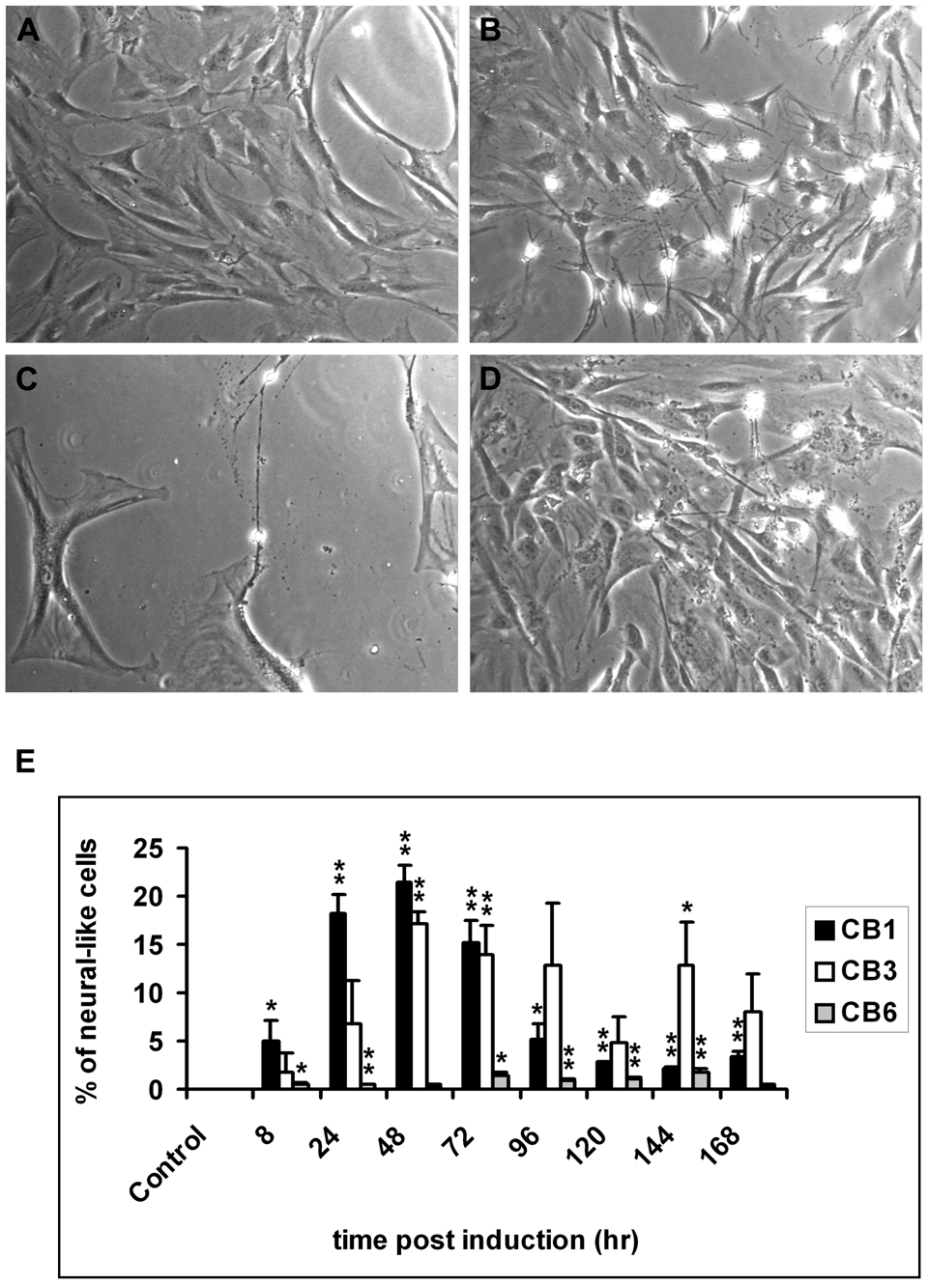
MSC^hUCBs^ respond with neurite-like extensions upon exposure to differentiation medium. (A–D). Phase contrast images comparing neurite-like outgrowth of three independent MSC^hUCB^ populations (MSC^hUCB1^, MSC^hUCB3^, and MSC^hUCB6^) following exposure to differentiation medium for 72 h (B, C and D, respectively), as compared to the undifferentiated control of MSC^hUCB1^ (A). (E) Quantification of neurite-like extensions for all three MSC^hUCBs^. Note that all MSC^hUCB^ populations responded with neurite-like outgrowth within 8 hours of incubation in differentiation medium, although to different degrees. MSC^hUCB1^ and MSC^hUCB3^ generated cells with neurite extensions, although MSC^hUCB1^ responded more efficiently. MSC^hUCB6^, on the other hand responded slower and with less efficiency. Experiments were carried out in triplicates and data are expressed as mean ± standard error of the mean (SEM). Statistical analysis was done using Student's t-test. ** denotes statistically highly significant (p<0.01) and * denotes statistically significant (p<0.05) data.

MSC^hUCB^ incubated with cytokine induction medium (CIM) appeared neural-like. Neural morphology was distinguished from the normal cell morphology by a number of criteria, including the refractile appearance of the cells, the presence of either bipolar or multipolar neurite-like projections (with the projection at least equal to or exceeding the diameter of the soma) and the occasional presence of secondary extensions. We investigated MSC^hUCBs^ derived from three independent cord blood samples (MSC^hUCB1^, MSC^hUCB3^ and MSC^hUCB6^) and assayed the responses of these cell lines to the differentiation medium in terms of the frequency of neurite outgrowth as well as the dynamics of appearance/disappearance of neurite-like extensions over time. All three cells lines displayed cells with neurite-like extensions as early as 8 h into differentiation (Fig. 1E) with MSC^hUCB1^ showing the highest frequency at this point of time. In addition, MSC^hUCB1^ also showed the steepest increase of neurite bearing cells until 48 h whereas MSC^hUCB3^ and MSC^hUCB6^ increased at slower rates. At 72 h, when MSC^hUCB1^ cells started to demonstrate a decreasing frequency of neurite bearing cells, MSC^hUCB6^ began to show an increasing trend. MSC^hUCB3^ showed a decreasing trend at 120 h. The observed decline in the frequency of cells with neurite-like extensions in all three cell lines could be due to a toxic effect of the differentiation medium, certain component(s) in it or as a result of natural attrition of differentiated cells in cell culture. Generally, MSC^hUCB1^ was the most efficient responder to the differentiation medium (Fig. 1B) whereas MSC^hUCB3^ responded slower but reached similar levels of neurite-like bearing cells at a slightly later time point (Fig. 1C). In contrast, MSC^hUCB6^ did not show substantial dynamics over time and was also generally the weakest responder in terms of generating cells bearing neurite-like extensions (Fig. 1D). Taken together, although all three MSC^hUCBs^ showed some levels of neurite-like extensions as a reaction to the differentiation medium, MSC^hUCB1^ seemed to be the quickest responder with the highest frequency of neurite-like bearing cells.

**Figure 2:**
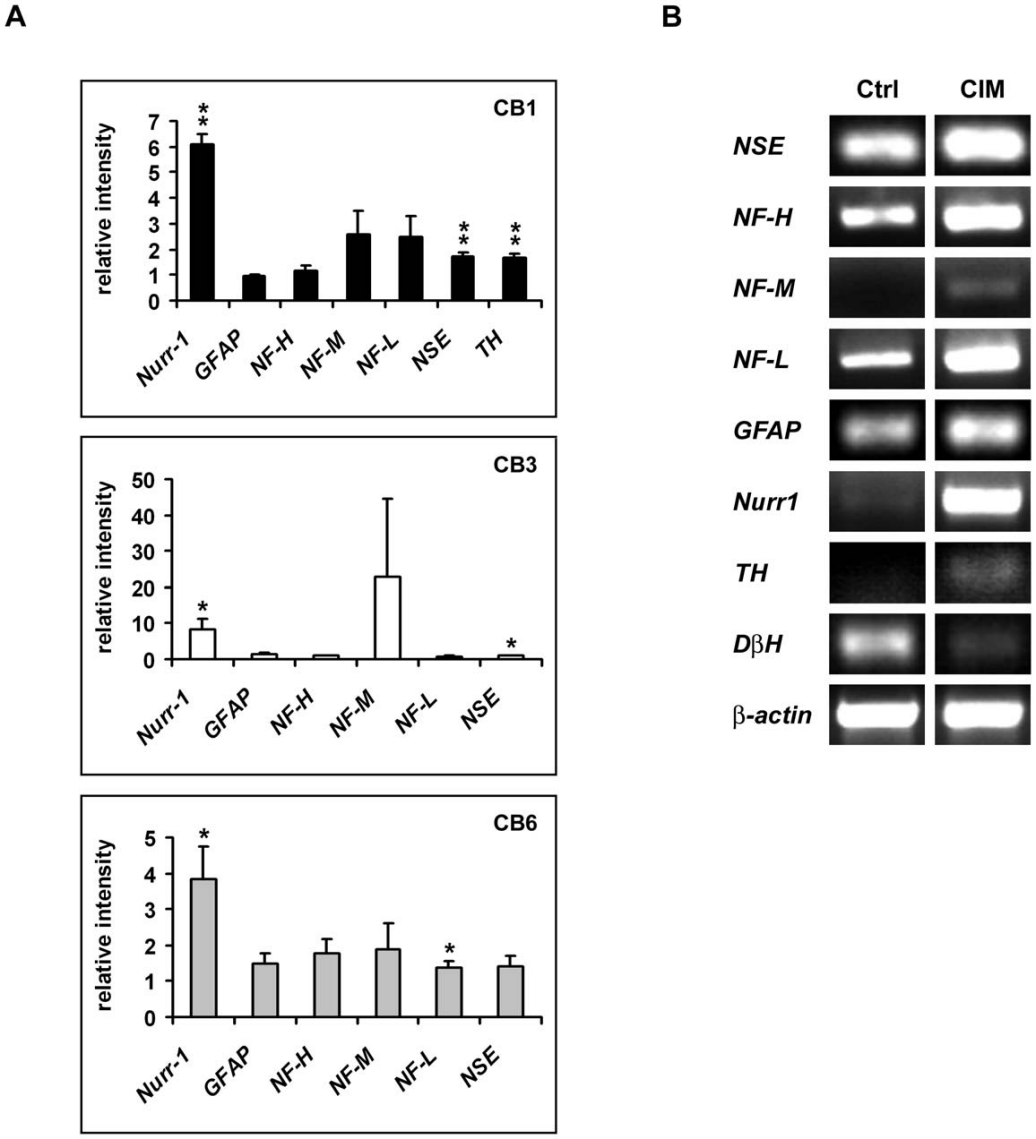
MSC^hUCBs^ upregulate neural specific markers. (A) Band quantification readouts of RT-PCR results of the three MSC^hUCBs^ after incubation in differentiation medium for 24 h, normalized to β-actin and the respective controls. Note that *Nurr1* upregulation is statistically significant in all the three MSC^hUCBs^. *NF-M* levels are also elevated, although not significantly. In contrast to MSC^hUCB3^, MSC^hUCB1^ and MSC^hUCB6^ could considerably upregulate other neuronal specific marker gene expression such as *NF-H* and *NF-L*, with MSC^hUCB1^ also showing upregulation of *TH* expression. Data are expressed as mean ± SEM (n = 3). (B) Gene expression profiling of MSC^hUCB1^ with RT-PCR. Representative RT-PCR products for each marker are shown. Neural markers such as *NSE*, *NF-H*, *NF-M*, *NF-L*, *GFAP* as well as the dopaminergic markers *Nurr1* and *TH* are upregulated following differentiation. Experiments have been performed in triplicates and data are expressed as mean ± SEM. Note the down-regulation of *dopamine-β-hydroxylase* (*DβH*) which encodes for the key enzyme that converts dopamine into nor-adrenaline.

It was shown that neurite-like extension can be induced by environmental signals such as stress [13], [14], [15], hence the presence of neurite-like extension alone is not conclusive for neural differentiation. We further investigated if the observed neurite extension was accompanied by regulation of neural marker gene expression by performing RT-PCR analysis. For all the three MSC^hUCBs^, levels of neuronal markers such as *Nurr-1* and *NF-M* were considerably upregulated (Fig. 2A). Although MSC^hUCB3^ could be quite efficiently induced morphologically, this particular cord blood cell line did not considerably upregulate neuronal marker gene expression such as *NF-H* and *NF-L*. Surprisingly, although MSC^hUCB6^ did not show much neurite-like outgrowth, it was able to upregulate neural marker gene expression (Fig. 2A). We did not detect meaningful differences in *TH* expression before and after incubation in CIM for MSC^hUCB3^ and MSC^hUCB6^ (data not shown). MSC^hUCB1^ was found to react best at upregulating marker gene expression when incubated with CIM. We observed that neuronal markers such as *neural specific enolase* (*NSE*), *neurofilaments* (*NF-H*, *NF-M* and *NF-L*) as well as the astroglial marker, *glial fibrillary acidic protein* (*GFAP*) were generally up-regulated in MSC^hUCB1^ (Fig. 2A, B). We also detected significant up-regulation of *Nurr1* and *TH*. In contrast, *dopamine beta hydroxylase* (*DβH*), the critical enzyme required to transform dopamine into noradrenalin was down-regulated during differentiation (Fig. 2B). In summary, of the three MSC^hUCBs^ tested, MSC^hUCB1^ responded most to the differentiation medium, as shown by the increased frequency of neurite-like outgrowth and upregulation of neural marker gene expression. Hence, we continued our further analysis with MSC^hUCB1^.

**Figure 3:**
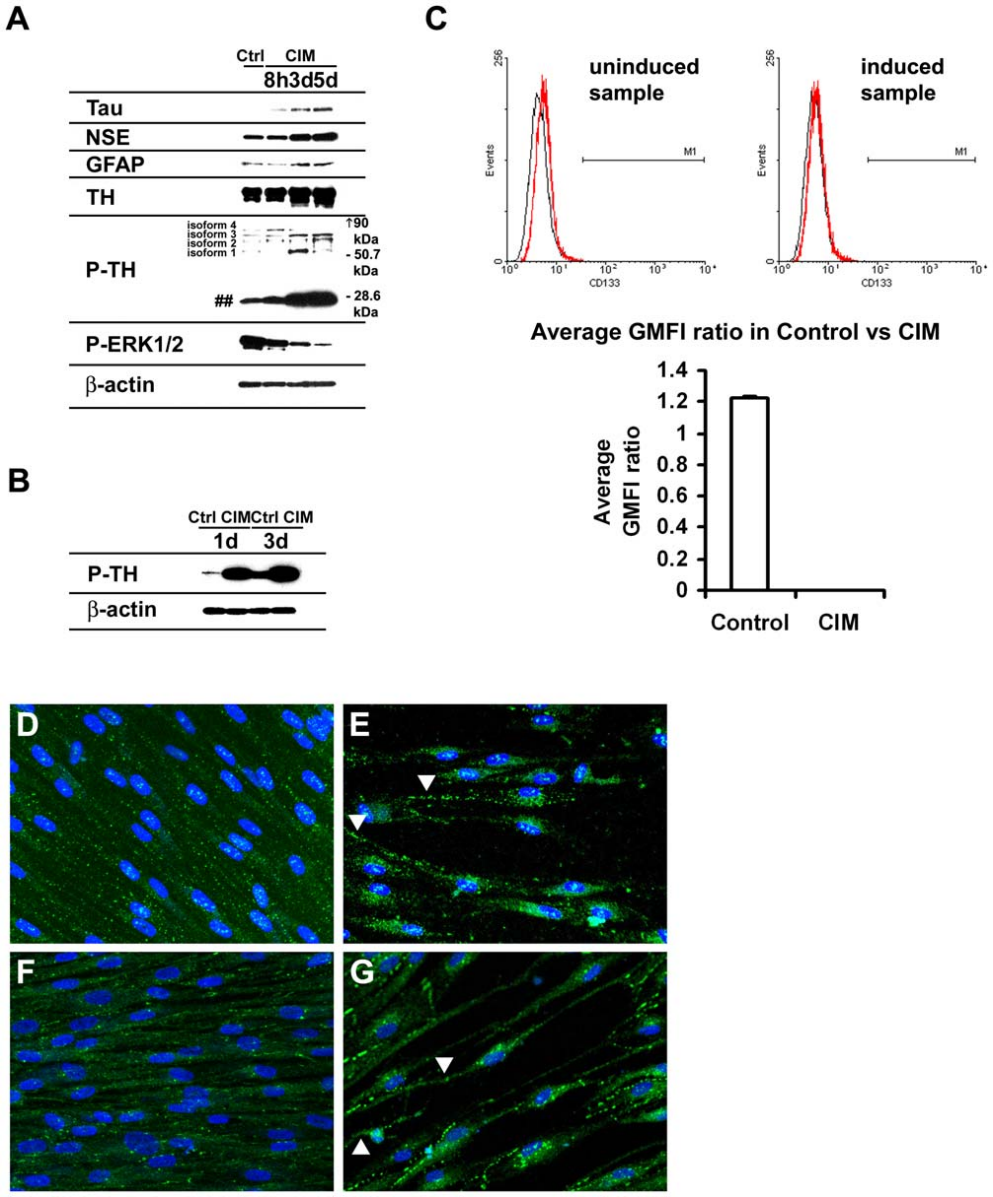
MSC^hUCB1^ express neural specific markers suggesting neural differentiation. (A) Protein expression profile. Expression of general neural markers (Tau, NSE and GFAP) and dopaminergic markers (TH and P-TH) are upregulated. The four P-TH isoforms are of sizes between 50.7 kDa and 90 kDa. Note that the different P- TH isoforms (isoforms 1, 3 and isoforms 2, 3) are differentially regulated (day 3 and day 5 post-induction, respectively). A small molecular weight band (∼28.6 kDa), possibly representing an unknown P-TH isoform is marked by ##. The MAPK pathway is down-regulated as a consequence of differentiation as shown by decreasing levels of P-ERK1/2 correlated to increasing length of differentiation. (B) The small molecular weight band (∼28.6 kDa) is strongly elevated in the dopaminergic SY5Y neuroblastoma cells in response to the differentiation medium. β-actin is used as loading controls (A, B). (C) Summary of the FACS analysis data showing the average geometric mean fluorescence intensity (GMFI) of CD133 expression of the experimental and the respective isotype control. CD133 is expressed more strongly in a small population of undifferentiated MSCs and weakly in the rest of the population. However, CD133 is completely down-regulated within 24 h of differentiation. Analysis was done in triplicates and data is expressed as mean ± SEM. (D–G) Immunocytochemical analysis of controls (D, F) versus differentiated MSC^hUCB1^ (E, G), showing cells stained with α-synaptophysin (Syp) (D and E) and α-P-TH (F and G). Cells were fixed after 3 days of incubation in differentiation medium. Note that Syp is strongly expressed at the projections (arrowheads in E). Although P-TH expression is already present at basal levels in the control, it becomes upregulated during differentiation. Arrowheads in G point to some cells with neurite-like extensions.

Exposure of MSC^hUCB1^ to CIM also led to upregulation of protein expression of neuronal markers such as Tau, NSE, a glial marker (GFAP) and the dopaminergic marker TH (Fig. 3A). TH has been known to exist as different isoforms as a consequence of differential splicing and phosphorylation has been shown to be a crucial step for enzymatic activation of TH [16]. We therefore assayed the phosphorylation of TH using a phospho specific TH antibody (p-Ser40) during differentiation. We found that MSC^hUCB1^ also expressed four P-TH bands of sizes between 50.7 kDa and 90 kDa, reflecting the previously described human TH isoforms [16], [17], [18]. Furthermore, we found that these isoforms were differentially regulated, with isoforms 1, 3 and isoforms 2, 3 being elevated on the third day and fifth day of induction, respectively. We also observed a small molecular weight band (∼28.6 kDa, Fig. 3A) which has not been described previously and might possibly represent an unknown P-TH isoform. Interestingly, this small molecular weight band was strongly upregulated following induction. A similar band was seen strongly upregulated during differentiation of the dopaminergic neuroblastoma cell line SY5Y (Fig. 3B) indicating that this band was not specifically upregulated in differentiating MSCs alone but also in the unrelated cell line SY5Y [19], [20].

The mitogen-activated protein kinase (MAPK) pathway has been associated with proliferation and differentiation [21]. Reduced activity of this pathway could be an indication of cells withdrawing from the cell cycle and progressing towards differentiation. We investigated the phosphorylation of extracellular signal-regulated kinases/ERK1/2 as a read-out of MAPK pathway activation status. We observed down-regulation of P-ERK1/2 with induction (Fig. 3A) demonstrating that neural differentiation was accompanied by down-regulation of the MAPK pathway and possibly an exit of the cell cycle. Previously, it was shown that the MAPK pathway was not involved in neural differentiation of MSC^hUCB^ [6].

CD133 expression has been linked to stem cells including MSCs [12]. We assayed MSC^hUCB1^ for the presence/absence of CD133 expression under control and differentiated conditions (Fig. 3C). We found that a small population of MSC^hUCB1^ cells expressed CD133 brightly (0.15%+/−0.015; (p<0.001)) in the undifferentiated MSC population. The remainder of the population also weakly expressed CD133 as indicated by a subtle but significant (p<0.05) shift in the average GMFI in the undifferentiated MSC population as compared to the isotype control. However, in the presence of differentiation medium, we neither observed the small fraction of cells that strongly expressed CD133 (0.06%+/−0.012; p>0.05 versus isotype staining) nor the shift in the average GMFI value of CD133. This suggests that CD133 expression was completely abolished in the differentiating cell population (Fig. 3C). In summary, our data reveal that the upregulation of neural marker gene expression during differentiation is accompanied by the down regulation of CD133 expression.

To further support that the MSC^hUCB^ showed differential marker expression in the differentiated vs. undifferentiated conditions which can be correlated with the observed morphological changes, we performed immunohistochemistry. We observed elevated levels of synaptophysin and phosphorylated TH (P-TH) in differentiating cells (Fig. 3D–E and Fig. 3F–G, respectively). Some of the responsive cells also showed neurite-like outgrowth, as indicated by the bipolar/multipolar extensions (arrowheads in Fig. 3E and 3G).

### IBMX and db-cAMP Are Required and Sufficient for Neurite Outgrowth

As the CIM medium was composed of a number of different components (see Materials and Methods), we dissected the medium to identify the components in the differentiation medium which were responsible for the observed outcomes. We started by investigating the effects of withdrawal of single components from CIM and addition of single components into the growth medium on neurite-like outgrowth of cells.

**Figure 4:**
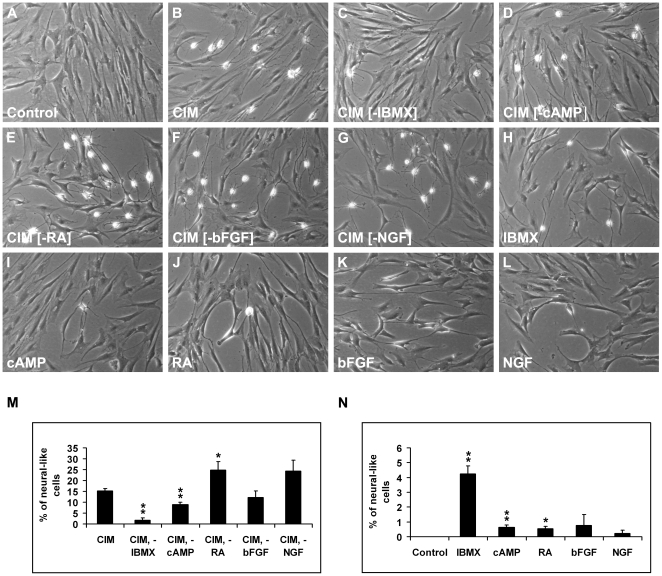
Analysis of neurite-like extensions of MSC^hUCB1^ in different media composition. (A) Control. (B) Cytokine induction medium (CIM). (C, D, E, F and G) Substraction of IBMX, db-cAMP, RA, bFGF and NGF from CIM, respectively. (H, I, J, K and L) MSC^hUCB1^ incubated with IBMX, db-cAMP, RA, bFGF and NGF, respectively. Note that IBMX and db-cAMP are necessary and sufficient for neurite-like outgrowth as their removal (C, D) resulted in less cells with neurite-like extensions and individually, (H, I) they induced neurite-like extensions. Removal of RA resulted in a higher frequency of cells with neurite-like extensions possibly due to a toxic effect of RA. If applied individually, RA resulted in small but statistically significant increase in cells with neurite-like extensions. bFGF and NGF did not have significant effects on neurite extension. (M) Quantification of cells with neurite-like outgrowth when individual components were removed from CIM. (N) Quantification of neurite-like extensions when MSC^hUCB1^ were incubated with single components. Data in (M) and (N) were analysed using Student's t-test. Experiments were performed in triplicates and data is expressed as mean ± SEM.

Induction of MSC^hUCB1^ with CIM resulted in ∼15% of cells with neurite-like outgrowth (15.3%+/−1.0, Fig. 4B, M). Removal of IBMX (1.8%+/−1.0, Fig. 4C, M) and db-cAMP (8.8%+/−1.3, Fig. 4D, M) resulted in highly statistically significant (p<0.01) fewer neural-like cells as compared to CIM. Therefore, IBMX and db-cAMP are required for neurite-like outgrowth. Removal of bFGF (12.1%+/−3.1, Fig. 4F, M) did not result in significant (p>0.05) changes in cells bearing neurite-like structures. Removal of RA (24.8%+/−3.9, p<0.05, Fig. 4E, M) or NGF (24.3%+/−5.0, Fig. 4G, M) resulted in more neurite-like processes, although removal of NGF did not give statistically significant results. When added individually to growth medium, IBMX highly significantly induced neural-like cells (4.3%+/−0.5, p<0.01, Fig. 4H, N), hence IBMX was sufficient to induce neurite-like extensions. db-cAMP and RA also induced neurite-like extensions significantly (0.6%+/−0.1 and 0.5%+/−0.1, respectively; p<0.05; Fig. 4I, J and N) but at a much lower percentage as compared to IBMX. When present alone bFGF and NGF did not induce neurite-like outgrowth (Fig. 4K, L and N).

### IBMX and db-cAMP Are Functionally Redundant for *Nurr1* Expression while IBMX and RA Synergistically Regulate *NF-L* Expression

**Figure 5:**
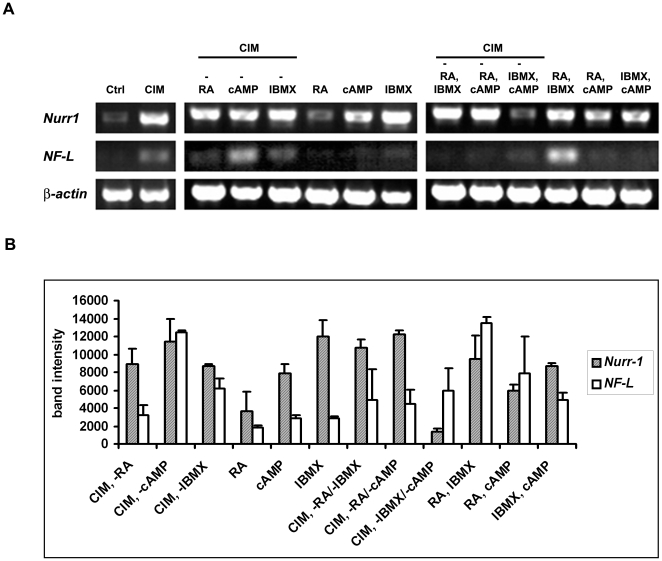
IBMX and db-cAMP upregulate *Nurr1* expression in a redundant manner and RA and IBMX synergistically regulate *NF-L* expression. (A) RT-PCR analysis showing mRNA expression of *Nurr1* and *NF-L* following different treatments. Note that IBMX and db-cAMP are sufficient for upregulating *Nurr1* expression. IBMX and db-cAMP have redundant roles as reflected by the reduction of *Nurr1* expression only in the absence of both components from the medium. RA and IBMX act synergistically on *NF-L* expression. (B) Quantification of RT-PCR results of (A). Experiments were performed in duplicates and data is expressed as mean ± SEM.

Since *Nurr1* and *NF-L* were upregulated in MSC^hUCBs^ when incubated with CIM, we were interested to identify the components that were responsible for the observed changes in gene expression. To reduce complexity, we excluded the two growth factors in our analysis and only focused on IBMX, db-cAMP and RA. In order to get more detailed insights into the overall changes, we also investigated the presence and absence of two media components. Analysis of transcriptional changes with RT-PCR showed that either IBMX or db-cAMP alone was sufficient to induce *Nurr1* expression. Consistently, removal of either IBMX or db-cAMP from CIM resulted in minimal changes on *Nurr1* expression. *Nurr1* transcription was strongly down-regulated only when both components were absent (Fig. 5), indicating that IBMX and db-cAMP had redundant roles on *Nurr1* transcription. In accordance with this observation, the presence of IBMX and db-cAMP in combination did not substantially elevate *Nurr1* levels over the levels of the individual components (Fig. 5). Unlike IBMX and db-cAMP, RA was not required for *Nurr1* transcription as its absence did not affect *Nurr1* expression and it was not sufficient to upregulate *Nurr1* (Fig. 5) when added to the cells. In fact, the presence of RA seemed to decrease levels of *Nurr1* expression. In the presence of RA, *Nurr1* transcription could be maintained only when either IBMX or db-cAMP was also present (Fig. 5).

Removal of either IBMX or RA resulted in down-regulation of *NF-L* expression, with removal of RA resulting in a stronger effect. Neither component alone was sufficient to elevate *NF-L* levels, however, in combination IBMX and RA strongly upregulated *NF-L* indicating their synergistic interactions on *NF-L* transcription (Fig. 5).

### RA or db-cAMP Results in Differential Phosphorylation of TH Isoforms

**Figure 6:**
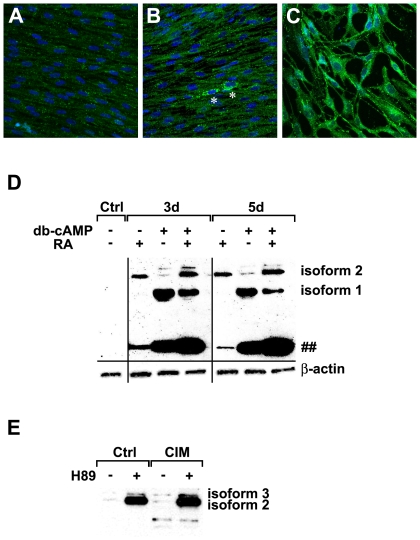
Immunocytochemical and biochemical analysis of P-TH expression. (A–C) Immunostaining with α-P-TH in (A) control, (B) RA and (C) db-cAMP treated MSC^hUCB1^. In general, RA elevates P-TH level with some cells expressing P-TH stronger (* in B). db-cAMP induces P-TH uniformly strongly in the entire cell population (C). (D) Western Blot analysis of P-TH expression. RA elevates P-TH isoform 2 and db-cAMP predominantly increases P-TH isoform 1. Presence of RA together with db-cAMP reduces the levels of P-TH isoform 1 which are highly elevated in the presence of db-cAMP, but only slightly increases the levels of isoforms 2. Note that the small molecular weight band (∼28.6 kDa, ##) is upregulated with induction, with the highest level of expression seen in the presence of both RA and db-cAMP. β-actin is used as the loading control. (E) Analysis of TH phosphorylation in the presence of a PKA inhibitor, H89, at 24 h post treatment. Note that incubation of MSC^hUCB1^ with H89 result in highly elevated levels of P-TH isoform 2 and slight increase in the levels of P-TH isoform 3, in both the controls as well as in the differentiation conditions.

We performed immunocytochemical and biochemical studies to investigate the roles of IBMX, db-cAMP and RA on TH phosphorylation. From immunostainings, we found that RA and db-cAMP were each sufficient to upregulate P-TH expression (Fig. 6A–C). A small population of RA treated cells could be seen to express TH more strongly although in general all the cells expressed higher level of P-TH as compared to the undifferentiated condition (Fig. 6A–B). Induction with db-cAMP was more efficient and resulted in more uniform TH expression in the entire population (Fig. 6C), whereas IBMX only minimally induced P-TH expression (data not shown). It is noteworthy that although TH expression seemed to be elevated when cells were incubated with either db-cAMP or RA, there were not many cells with neurite-like outgrowth (as also shown in Fig. 4I and J). To investigate if the immunocytochemical results were reflected by the change in total protein levels when incubated with the same set of components, we performed Western Blot analysis. We found that IBMX had minimal effects on total P-TH levels (data not shown). RA and db-cAMP both increased P-TH levels although RA induced phosphorylation of TH isoform 2 whereas db-cAMP predominantly induced isoform 1 (Fig. 6D). Consistent with the immunocytochemical data (Fig. 6B–C), treatment with db-cAMP seemed to result in higher levels of P-TH as compared to treatment with RA. When applied together, RA seemed to repress db-cAMP mediated upregulation of isoform 1 but expression of isoform 2 was only slightly increased (Figure 6D).

### The PKA Pathway Inhibits Phosphorylation of Two TH Isoforms

The PKA pathway is activated by the second messenger cAMP which in turn leads to the phosphorylation of the Cre-binding protein (CREB) and modulation of transcription of downstream genes [22], [23]. Previously, we reported that the PKA pathway contributed to aspects of neural differentiation of MSC^hUCB^ [6]. Both IBMX and db-cAMP have been known to increase intracellular cAMP concentrations, which may result in the activation of the PKA pathway and TH phosphorylation [16], [24]. We investigated if the PKA pathway was also involved in TH phosphorylation under our experimental regime. We co-incubated MSC^hUCB1^ cells with the PKA inhibitor H89 followed by analysis of protein levels with the α-P-TH antibody. We observed highly elevated levels of P-TH isoform 2 following H89 treatment in the presence as well as in the absence of induction (Fig. 6E). Slightly higher levels of P-TH isoform 3 were also observed but levels of P-TH isoforms 1 and 4 remained largely unaffected. This suggests that PKA acted as a negative regulator of phosphorylation for at least one TH isoform.

## Discussion

It was shown previously that when exposed to defined cell culture conditions, cord blood derived mesenchymal stem cells were capable of acquiring neural-like characteristics [4], [6], [25]. Here, we studied the effect of a defined induction medium on neural differentiation as well as phosphorylation of TH, the gold standard marker for DA neurons. We also examined the effects of individual medium components and combinations thereof in order to address the possible underlying molecular and biochemical mechanisms involved in neural differentiation.

### MSC^hUCBs^ Undergo Neural Differentiation and Express Markers Associated with Dopaminergic Neurons

We initially analyzed three MSC populations which were derived from three individual donors. Although all three MSC populations reacted to the induction medium by extending neurites and showing elevated levels of neural marker genes, MSC^hUCB1^ was generally the most responsive in inducing neurite outgrowth and upregulating neural specific marker gene expression such as *NSE*, *neurofilaments*, *GFAP*, *Nurr1* and *TH*. The three *neurofilament* genes (*NF-L*, *NF-M*, *NF-H*) were differentially expressed in the undifferentiated and differentiated conditions, possibly as a consequence of being independent transcription units and hence were differentially regulated [26]. In contrast to TH protein which was already expressed in undifferentiated MSC^hUCB1^, *TH* mRNA was undetectable maybe due to it being less abundant or relatively unstable, possibly in combination with a long half life of the TH protein.

TH contains serine residues which are phosphorylated by a number of kinases [27] leading to its enzymatic activation [16]. We found that phosphorylation of distinct TH isoforms changed with length of exposure to CIM, suggesting time dependent and differential TH phosphorylation. In addition to the four reported TH splice variants [16], [18], we detected a small molecular weight band which may represent a novel phosphorylated TH isoform. This band was also up-regulated in the induced neuroblastoma cell line SY5Y, suggesting that its presence and upregulation under differentiation conditions might be a more general feature which is not restricted to MSCs.

Although TH is the rate limiting enzyme for dopamine (DA) synthesis, DA is the precursor for norepinephrine and the conversion of DA into norepinephrine requires Dopamine-β-hydroxylase (DBH). Hence, the presence of both enzymes in the same cell would indicate an adrenergic rather than a dopaminergic cell fate [28]. Interestingly, in conjunction with *TH* upregulation we observed down-regulation of *DBH* expression with differentiation, suggesting that differentiating neurons could rather adopt a dopaminergic phenotype. Overall, our data suggest that some crucial components of the genetic network leading to neuronal fate as well as the molecular machinery required for post-translational modification of the TH protein are present in differentiating MSC^hUCB1^. Furthermore, the elevated levels of neural marker gene expression seen in the differentiated MSC^hUCB1^ was accompanied by the loss of a marker associated with undifferentiated MSCs, suggesting that MSC^hUCB1^ could be differentiated to some degrees towards neural fate. However, whether such cells are bona-fide and functional neurons exhibiting mature neurotransmitter phenotypes requires further careful analysis. On the other hand, it was reported that under similar experimental conditions, cord blood derived stem cells were capable of producing and releasing dopamine into the differentiation medium [7] suggesting that such cells were at least partially functional DA neurons.

### Roles of Different Media Components on Neurite Outgrowth, Gene Regulation and TH Phosphorylation

In order to gain insights into the molecular and biochemical mechanisms underlying neural differentiation of MSC^hUCB^, we examined the roles of individual as well as combinations of components from CIM for processes such as neurite-like outgrowth, marker gene expression (*Nurr1* and *NF-L*) and TH phosphorylation.

IBMX and db-cAMP increase levels of intracellular cAMP which can result in PKA activation [22]. cAMP modulates neural differentiation [29], neuroendocrine differentiation [30], neuronal differentiation of glioma cells [31], [32], neurite like outgrowth in medulloblastomas [33] and neural differentiation of bone marrow MSC [34]. Activation of PKA as well as PKC agonists were known to result in DA differentiation [35]. In our experiments, IBMX and db-cAMP were both required for morphological differentiation of MSC^hUCB1^. Both components induced neurite-like extension of MSC^hUCB1^, although the efficiency of db-cAMP was lower. Our data suggests that neurite extension might be caused by an increase of intracellular cAMP levels possibly resulting in the activation of the PKA pathway. This is consistent with a report on a role for PKA in regulating neurite outgrowth in a variety of neuronal cell lines [36]. IBMX and db-cAMP were also sufficient and redundant in the upregulation of *Nurr1* expression. Previously, we have shown that the PKA signaling pathway triggered by forskolin was also involved in the regulation of *Nurr1* expression during MSC differentiation [6].

RA is an active derivative of vitamin A. Its wide expression in the nervous system during development, its role as a potent inducer of cell differentiation and the wide expression of its receptors and binding proteins in the brain suggest an important role of RA in brain development and function [37]. Nurr1 heterodimerizes with retinoic acid receptors of the RXR class, contributing to DA survival [11]. Nurr1 and RA were also reported to induce cell cycle arrest in dopaminergic MN9D cells which was accompanied by a highly differentiated state of cells with long neurite extensions [38]. Hence, RA and Nurr1 might play important roles in the differentiation and maintenance of DA neurons. We found that RA was neither required nor sufficient for inducing neurite-like outgrowth. RA was also unable to activate *Nurr1* expression in MSC^hUCB1^ and it had limited effects on *NF-L* expression; however, the combination of IBMX and RA induced *NF-L* expression, suggesting synergistic interactions between both for *NF-L* expression. As such, we propose that *NF-L* expression requires the activation of the cAMP/PKA pathway as well as some other pathways linked to RA.

Despite its inability to induce neurite outgrowth and *Nurr1* expression, RA was sufficient to increase phosphorylated TH levels in MSC^hUCB1^. On the cellular level, we found that a small number of RA-induced cells expressed higher levels of P-TH. This limited response of individual cells could be due to the heterogenous nature of the cell population. We also found that TH isoforms were differentially phosphorylated when MSC^hUCB1^ were treated with either RA or db-cAMP. TH isoforms are differentially expressed in the human brain [39] and it is conceivable that RA and db-cAMP activate distinct signaling pathways and currently unidentified down-stream effectors which might eventually contribute to differential phosphorylation of TH isoforms. It is noteworthy that although treatment with RA and db-cAMP increased levels of phosphorylated TH, cells did not respond with obvious neurite outgrowth. This indicates that neural marker gene expression and neurite outgrowth might be two uncoupled events triggered by different molecular mechanisms.

We also provide some evidence that the PKA signaling pathway might have a suppressive role in the phosphorylation of at least one TH isoform, as indicated by the increased levels of P-TH in the presence of a PKA inhibitor. Previously, it was reported that inhibition of the PKA pathway led to decreased levels of P-TH [40], which suggested an inductive role of PKA in TH phosphorylation. In the context of the observed suppressive role, PKA could either inactivate a down-stream kinase involved in TH phosphorylation or activate a phosphatase that dephosporylates TH. Inhibitory mechanisms for TH phosphorylation have been described in striatal neurons in which activated D2-receptors resulted in a reduction of Ser40 phosphorylation of TH [41]. However, the precise molecular and biochemical mechanisms involved in the regulation of TH activity require further investigations.

Recently, reports raised concerns that neural differentiation of MSCs might be a reflection of cellular stress rather than cells entering a true differentiation programme [13], [14], [15]. Based on our results, we suggest that MSC^hUCBs^ can be triggered to express neural marker genes as a consequence of differentiation rather than stress: firstly, the studies linking neural differentiation of MSCs to cellular stress were done with bone marrow derived MSCs. Similar studies have not been performed with cord blood derived MSCs. Secondly, our observations of morphological changes were accompanied by elevated expression of general neural markers as well as markers which are known to be active in differentiation programs towards specific neurotransmitter phenotypes. This suggests the activation of defined molecular programmes related to differentiation. Thirdly, our data indicate an activation of a biochemical programme involved in post-translational modification of a neural specific gene, *TH*. Fourthly, we found that particular components of CIM differentially up-regulated different P-TH isoforms. In addition, some components of the differentiation medium were required synergistically to regulate marker genes. Together, these findings suggest that cells responded specifically to defined stimuli. Finally, it was reported that neural differentiated cells from cord blood using similar experimental conditions resulted in release of the neurotransmitter DA combined with the acquisition of electrophysiological properties reminiscent at least of immature neurons [7], [42]. Thus at least partially functional neurons were generated.

Taken together, we have shown that our experimental paradigms activated complex molecular and biochemical programs leading to multifaceted changes of properties of cells towards neuronal characteristics. Whether differentiation programmes leading to MSCs neural differentiation share common mechanisms with differentiation of primary neurons during development or differentiation of neural stem cells *in vitro* remains to be determined. Our findings also emphasize on the requirement of detailed knowledge of the molecular and cellular mechanisms of neural differentiation of MSCs in order to direct stem cells towards the neuronal fate and possibly to specific neurotransmitter phenotypes. The possibility of differentiating MSCs from cord blood into cells expressing markers associated with DA neurons and further mechanistic insights into achieving this *in vitro* with high efficiency might facilitate the generation of fully functional DA neurons in the future that could be suitable for cell replacement therapies in neurodegenerative diseases such as PD.
